# Efficacy and Safety of Transcranial Magnetic Stimulation for Treating Late-Life Depression: A Scoping Review

**DOI:** 10.3390/jcm14103609

**Published:** 2025-05-21

**Authors:** Ciprian-Ionuț Băcilă, Monica Cornea, Andrei Lomnasan, Claudia Elena Anghel, Andreea Maria Grama, Cristina Elena Dobre, Silvia Rusu, Bogdan Ioan Vintilă

**Affiliations:** 1 “Dr. Gheorghe Preda” Clinical Psychiatry Hospital of Sibiu, 550082 Sibiu, Romania; ciprian.bacila@ulbsibiu.ro (C.-I.B.); claudia.anghel@ulbsibiu.ro (C.E.A.); andreeamariaps@gmail.com (A.M.G.); silviarusu@yahoo.com (S.R.); 2 Faculty of Medicine, Lucian Blaga University of Sibiu, 550169 Sibiu, Romania; bogdan.vintila@ulbsibiu.ro; 3 Neuroscience Scientific Research Collective, 550082 Sibiu, Romania; 4 Socola Institute of Psychiatry, 700282 Iasi, Romania; cedobre@yahoo.com; 5 County Clinical Emergency Hospital of Sibiu, 550245 Sibiu, Romania

**Keywords:** TMS, geriatric depression, efficacy, safety

## Abstract

**Background/Objectives**: Transcranial magnetic stimulation (TMS) is a non-invasive and well-tolerated treatment, offering an effective alternative for elderly patients with depression, especially when side effects or comorbidities limit medication. **Methods**: This scoping review analyzes 16 studies published over the past seven years, to evaluate the efficacy, safety, and clinical applications of TMS in older adults with depression. **Results**: The review examines various TMS modalities, including repetitive TMS (rTMS), deep TMS, and theta burst stimulation (TBS), with most protocols targeting the dorsolateral prefrontal cortex (DLPFC). Adverse effects were rare, mild, and transient, supporting the treatment’s safety profile. Pharmacological co-treatment was common but not essential for clinical improvement, highlighting TMS’s potential as a standalone therapy. A subset of studies used neuroplasticity (SICI, ICF, CSP) or neuroimaging measures (MRI and MRI-based neuronavigation), revealing that age-related cortical inhibition may limit plasticity rather than depression itself. **Conclusions**: Overall, TMS demonstrates promising effectiveness and tolerability in managing late-life depression. Across studies, remission rates varied from 20% to 63%, with higher efficacy generally observed in bilateral stimulation or high-frequency protocols. Standardization of protocols and further research into individualized targeting and long-term outcomes are warranted to support broader clinical adoption.

## 1. Introduction

Transcranial magnetic stimulation (TMS) uses a coil placed at the cortical level to generate an electric field that leads to neuronal depolarization [1,2]. rTMS applies repetitive pulses for 15 to 30 min, with pulse intensity ranging between 80% and 120%. The standard protocol includes five sessions per week for 4 to 6 weeks. High frequencies of 10 Hz or low frequencies of 1 Hz can be used [1]. The advantages of TMS are rare adverse reactions, better tolerance compared to ECT [1,3], no need for anesthesia [3], and effectiveness even in treatment-resistant depression [1]. The disadvantages are the high cost and the need for significant patient commitment, requiring at least one hour per day, five to six days per week. The elevated cost of TMS stems from the requirement for advanced equipment and the extensive training necessary for appropriately skilled personnel [1]. Since 2008, it has been approved as a treatment indication for major depressive disorder resistant to pharmacotherapy [2,4]. Studies have shown that TMS application reduces suicidal ideation and shortens the duration of pharmacological treatment [5].

Depression in the elderly occurs in approximately 15% of adults over 65 years [6,7], being a disabling medical condition comparable to cardiovascular diseases and diabetes [8,9]. Depressive symptoms affect both quality of life and social functionality [6]. Risk factors include medical conditions (cerebrovascular diseases, cognitive decline, other chronic illnesses specific to aging) and psychological and social factors (loneliness, loss of a life partner, social isolation) [10,11].

Geriatric depression frequently coexists with a multitude of chronic medical conditions, including cardiovascular disease, diabetes mellitus, neurodegenerative disorders, and chronic pain syndromes. This intricate interplay between depressive symptomatology and somatic illness often exacerbates functional decline, impairs treatment adherence, and diminishes overall quality of life in elderly individuals. Moreover, late-life depression is not merely a psychological affliction; it is a multifaceted clinical entity that amplifies morbidity and mortality, both through direct neurobiological mechanisms and indirect behavioral pathways. Recognition and management of comorbid conditions are thus imperative in addressing the full scope of geriatric depression, necessitating a comprehensive, interdisciplinary approach to care [10,11].

Depression treatment can be both pharmacological and non-pharmacological. Medication includes antidepressants, and the specialized literature presents studies showing that antidepressant use also has a beneficial effect on cognitive functions [10,12,13]. Also, the findings of Maina et al. (2023) [14] are relevant to geriatric depression, where treatment resistance is common due to age-related factors. Their emphasis on individualized strategies and the use of s-ketamine in outpatient settings supports the development of tailored approaches suitable for older adults with TRD [14]. Other methods for treating depression in the elderly include psychotherapy, especially cognitive-behavioral therapy, maintaining an active lifestyle, electroconvulsive therapy [8,15], and transcranial magnetic stimulation [5]. Elderly patients may not respond optimally to antidepressant medication, and therapeutic options are further limited due to somatic comorbidities, which increase the risk of adverse reactions. This results in restrictions such as drug selection and the use of lower doses [5,16,17]. RTMS can be used as an alternative treatment method for geriatric depression, being a safe and non-invasive approach [5]. It works by stimulating neuronal activity and has broad applicability in psychiatric pathologies.

This review primarily aims to analyze other specialized studies on the effect of transcranial magnetic stimulation in treating geriatric depression. This study employed a scoping review methodology outlined by Arksey and O’Malley [18] to map the existing literature on transcranial magnetic stimulation (TMS) for late-life depression. Given the heterogeneity in TMS modalities (e.g., rTMS, deep TMS, theta burst stimulation), stimulation parameters, outcome measures, and population characteristics, a scoping review was more appropriate to identify key concepts, types of evidence, and knowledge gap, and it is particularly suited for emerging areas of research with complex and diverse study designs, as is the case with neuromodulation in geriatric psychiatry.

In geriatric patients diagnosed with depression, transcranial magnetic stimulation (TMS) has emerged as a promising treatment modality, especially when side effects or polypharmacy concerns may limit traditional pharmacotherapy. There is a need to evaluate the efficacy of various transcranial magnetic stimulation (TMS) protocols and neurophysiological measures like EEG, functional MRI evoked potentials, EMG; therefore, this study seeks to address the following research question: among geriatric patients diagnosed with depression, how do different TMS protocols and neurophysiological assessments compare in their effectiveness for evaluating cognitive function and improving clinical outcomes?

## 2. Materials and Methods

### 2.1. Literature Search Strategy

A total of 102 studies were analyzed, of which 16 were included and 86 were excluded. The databases used for study selection were PubMed, Web of Science, UpToDate, and Scopus. Only studies conducted within the past 7 years were considered for inclusion. This timeframe was chosen to ensure the relevance and timeliness of the findings while also reflecting recent methodological advancements. Furthermore, studies published within the past seven years are more likely to reflect current diagnostic criteria, treatment guidelines, and methodological standards, thereby enhancing the relevance and applicability of the findings to contemporary clinical practice.

The studies reviewed encompassed observational and interventional designs, including both randomized and non-randomized trials, and were conducted prospectively.

Three authors, CIB, MC, and AL, conducted the literature search and article screening process. Disagreements that arose during the study selection were resolved through a consensus-based approach. Reviewer discrepancies were discussed in detail to ensure a mutual understanding of inclusion criteria and data extraction protocols. If consensus could not be reached, a third independent reviewer was consulted to make the final determination. This approach ensured the reliability and objectivity of the selection process, minimizing bias and enhancing the rigor of the review.

The key terms used in the analysis of the studies are as follows: late-life depression, geriatric depression, TMS, and efficacy.

### 2.2. Study Selection Process

The inclusion criteria were as follows: studies analyzing the effect of transcranial magnetic stimulation (TMS) on geriatric depression over 65 years, studies providing data on patient outcomes following the completion of TMS sessions, studies published in English, and studies published within the last 7 years. Observational studies and interventional clinical studies. The exclusion criteria were the following: studies that did not examine the impact of transcranial magnetic stimulation on geriatric depression, studies focusing on the effects of TMS on other psychiatric conditions specific to older adults, studies published in languages other than English, and studies published more than 7 years ago.

The analyzed data from the studies include the following: the effectiveness of TMS and how it was quantified (subjectively, through psychological scales, or functional methods), the application method of TMS, as well as session characteristics such as session duration and number of sessions, identification of adverse reactions, the concurrent use of pharmacological treatment, and the specific therapies administered.

It is important to acknowledge that a formal quality assessment was not conducted for this scoping review. While the studies included were systematically selected based on predefined inclusion and exclusion criteria, the main objective was to explore the extent and diversity of the existing literature rather than to evaluate the methodological quality of the individual studies. Consequently, this review does not include an evaluation of study design, risk of bias, or the robustness of findings.

## 3. Results

Sixteen studies, all original research studies, were analyzed. Their characteristics are presented in Table 1. The studies were selected using the PRISMA criteria.

**Figure 1:**
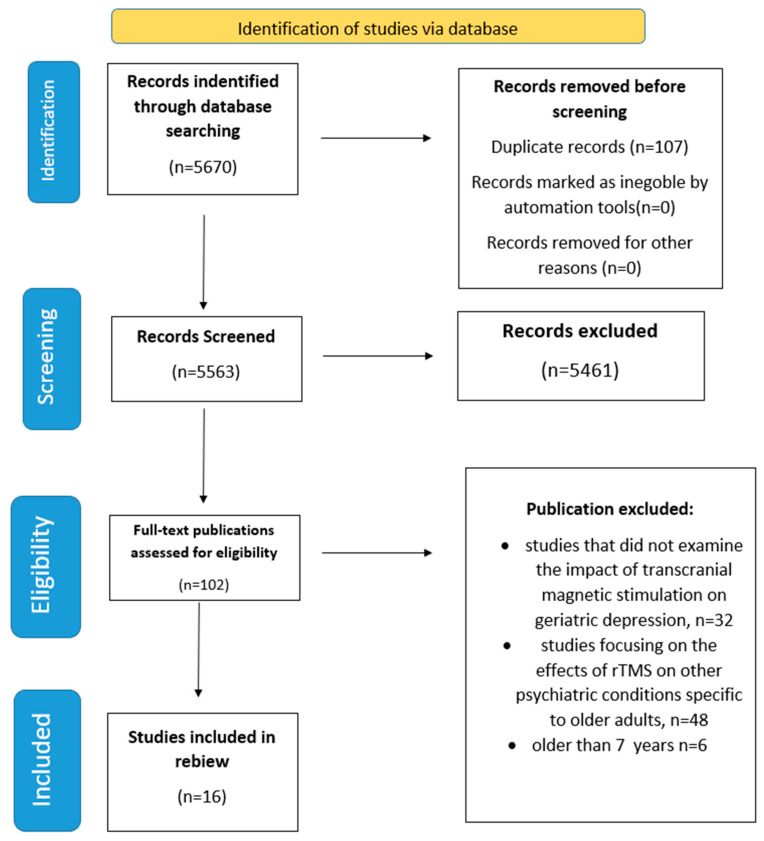
The literature search PRISMA flow diagram.

In this scoping review, the Preferred Reporting Items for Systematic Reviews and Meta-Analyses Extension for Scoping Reviews (PRISMA-ScR) guidelines were followed to ensure a systematic and transparent approach. These guidelines were applied to provide clear reporting on the identification, selection, synthesis of studies, and the methodology used throughout the review process (Figure 1).

### 3.1. Adverse Reactions

According to the studies analyzed, no adverse reactions were identified at the end of TMS sessions. In nine studies, adverse reactions were identified. A common observation is that adverse reactions do not have a long-term impact on the patient, as they are typically transient and minor [22,27,34]. Patients have subjectively reported the primary adverse reaction, which is predominantly pain on the right side in the case of TMS, where the stimulation was performed continuously for 40 s. However, the effect was not sustained in the long term, and this situation did not deter patients from discontinuing treatment [21,26,31]. Other adverse reactions reported were sleep disturbances, headache, and scalp discomfort [28].

After analyzing the studies, it was observed that TMS is a safe procedure, with rare side effects. When these side effects do occur, they are of minor intensity and transient, having no significant impact on the patient.

### 3.2. Association with Pharmacological Treatment

Of the 16 studies analyzed, 10 included pharmacological therapy in conjunction with TMS. The majority specified the class of medications used, while two studies provided details on the exact pharmacological agent, and three did not disclose the medication administered. The primary drug classes employed were antidepressants, antipsychotics, benzodiazepines, mood stabilizers, and hypnotics [20,21,26,30,31]. The overall findings were predominantly favorable, indicating that the combination of medication and TMS contributed to symptom improvement and enhanced outcomes in psychological assessments [20,21,30,31]. However, one study reported no significant efficacy for this combined approach [20]. In studies where pharmacological therapy was not administered concurrently, the outcomes remained positive, with observed improvements in depressive symptoms [22,25,28,29,32,33].

### 3.3. Efficacy and TMS Protocols

This review examines various types of TMS as treatment modalities, including TMS/rTMS, deep TMS, and TBS. The protocols associated with these modalities were also analyzed to assess their relative clinical effectiveness, as described in the included studies.

#### 3.3.1. Efficacy and TMS/rTMS Protocols

The studies included in this review employ protocols specifically designed to address distinct research questions, with methodological variations in device selection, stimulation parameters, and targeted brain regions. As outlined in Table 2, the studies utilizing TMS or rTMS have their stimulation parameters analyzed in detail within the table. Since some studies used the same participant sample as other studies [26,31], those articles were excluded from consideration for inclusion in this section. Additionally, Alheri’s study notes that the analyzed population exhibits significant heterogeneity in treatment protocols. As a result, this study is not included in this section.

The majority of studies utilize devices primarily from Magstim and MagVenture, with some incorporating additional systems such as Theracell. In terms of target areas, as seen in Table 3, most protocols focus on the dorsolateral prefrontal cortex (DLPFC), either unilaterally (left) or bilaterally, while a smaller subset targets the motor cortex or other specific brain regions.

Regarding stimulation parameters, frequencies range from low (1 Hz) to high (20 Hz), with intensities commonly determined based on the resting motor threshold (RMT). Low frequency (3 Hz) is used primarily in bilateral stimulation and inhibitory protocols (13), high frequency (10–20 Hz) is generally associated with excitatory effects and used in unilateral stimulation [25,30,32], while mixed approaches (1 Hz + 10 Hz) in different areas) is used for differential effects on brain regions [21]. Taking intensity into consideration, most studies use 110–120% RMT, which aligns with standard therapeutic protocols in clinical practice. Some studies employ adjustable intensity, particularly for protocols focusing on motor cortex excitability [20,23]. Certain protocols [20,23,24,31] emphasize specific stimulation paradigms such as short-interval intracortical inhibition (SICI), intracortical facilitation (ICF), paired associative stimulation (PAS), or cortical silent period (CSP), and are focused on neurophysiological assessments. In terms of treatment duration, individual sessions typically last between 15 and 30 min, with a total of 15 to 30 sessions. Some protocols described a progressive modification of the number of sessions, based on remission status [21,25,32], while Jodoin [30] described a more frequent stimulation (twice daily).

Regarding remission rates, 6 of the 10 studies included in this section did not report data on remission rates. However, considerable variability was observed among those that did. Jodin et al. [30] reported the highest remission rate at 63%, while Blumberger et al. [21] documented a remission rate of 32.9%. Trevizol et al. [32] found a 40% remission rate following bilateral rTMS, whereas no remissions were observed with unilateral stimulation. Leuchter et al. [25] reported remission rates ranging from 25% to 27%, depending on the specific psychological assessment scale employed.

#### 3.3.2. Efficacy and Deep TMS Protocols

Two studies included in this review, Roth et al. [29] and Kaster et al. [29,34], investigated deep transcranial magnetic stimulation (TMS) as a therapeutic approach. Roth et al. utilized two different stimulator devices (Magstim Rapid2 or BrainsWay 104), whereas Kaster et al. [34] exclusively employed the BrainsWay system. Both studies used the BrainsWay H1 coil; however, Kaster et al. initially implemented the H1L coil but discontinued its use due to poor tolerability, which resulted in participant dropouts and adverse effects, such as pain and seizure. Kaster et al. administered a significantly higher total number of pulses (6012) compared to Roth et al. (1980) and conducted longer treatment sessions (61 min vs. 20 min) [34]. Despite these differences, both protocols maintained the same stimulation frequency (18 Hz) and intensity (120% of the resting motor threshold). In terms of target regions, Roth et al. [29] focused on the left dorsolateral prefrontal cortex (DLPFC), whereas Kaster et al. stimulated both the DLPFC and ventrolateral prefrontal cortex (VLPFC) bilaterally, with a greater emphasis on the left hemisphere [34]. Regarding remission rate, Kaster [34] provided a rate of response for active deep rTMS of 44.0% compared to sham rTMS, 18.5%, while Roth [29] provided a response rate of approximately 70% after 20 sessions and 80% after 30 sessions, and a remission rate of 40% after 20 sessions and 60% after 30 sessions. Regarding response rates, Roth described an 89% response rate after 30 sessions and a 73% response rate after 20 sessions, while Kaster reported a 44–55% response rate.

#### 3.3.3. TBS Protocols

Although four studies were considered for this section, only two articles’ data related to theta burst stimulation (TBS) parameters were analyzed. This is because two of the included articles, Wathra [31] and Goke [26], utilized the same population and protocol from the Blumberger [21] study, thereby representing duplicate data. Although Blumberger’s study used both repetitive transcranial magnetic stimulation (rTMS) and theta burst stimulation (TBS), only data derived from the application of TBS were considered for this section of the review. In Almheiri’s study referenced in this review, both TBS and ITBS are discussed; however, the specific application of these methods to the geriatric population is not thoroughly detailed. Therefore, this article will be excluded from this section [19].

In the four studies included in this review, theta burst stimulation (TBS) was utilized as a form of transcranial magnetic stimulation (TMS). Blumberger [21] described the treatment protocol as bilateral TBS, with continuous TBS (cTBS) administered over the right dorsolateral prefrontal cortex (DLPFC), consisting of 600 pulses delivered over 40 s. In contrast, intermittent TBS (iTBS) was applied over the left DLPFC, comprising 600 pulses over 3 min and 9 s, with a stimulation pattern of 2 s on and 8 s off. Quinn [28], in his study, reported delivering TMS as iTBS over the right DLPFC, with each session consisting of 1800 pulses (60 trains of 10 triplet bursts), following the standard iTBS protocol (2 s on, 8 s off). In both studies, the MagPro X100 device with a B70 fluid-cooled coil (MagVenture) was used. The stimulation intensity was set at 120% of the resting motor threshold (RMT) [21,28].

Regarding the treatment schedule, Blumberger [21] described an initial protocol of 20 daily sessions over four weeks, with the possibility of an additional 10 sessions in cases where remission was not achieved, resulting in a maximum of 30 sessions. Quinn, on the other hand, employed an accelerated protocol consisting of 45 total sessions, delivered as five sessions per day over nine weekdays [28].

Regarding the remission rate, Blumberger [21] provided a 35.4% rate, while Quinn [28] documented a 20% rate. Blumberger reported a 41.9–44.3% response rate, and Quinn reported a 52% response rate.

### 3.4. Efficacy and Neuroplasticity Measurement

Out of the 16 studies that were analyzed in the review, only four evaluated neuroplasticity in using TMS for treating depression in the geriatric population. Two of the studies had additional group comparisons: Bhandari [20] used age-matched healthy controls, while Lissemore [23] used age-matched and unmatched healthy controls and younger patients with depression (only information concerning older adults with depression was considered). Bhandar [20] and Lissemore [23] explicitly measure PAS-induced plasticity, while Lissemore [23] and Wathra [33] focus on inhibition, which indirectly affects plasticity, as seen in Table 2. In all four studies, the cortical target was the left motor cortex. For PAS, Bhandar and Lissemore et al., and Wathra used proper median nerve stimulation, targeting sensorimotor pathways. In all three studies, the nerve stimulation is paired at 25 25 ms interval with TMS over the left motor cortex to probe LTP-like plasticity [20,23,31].

Short-interval intracortical inhibition (SICI), cortical silent period (CSP), and intracortical facilitation (IF) were considered in Lissemores [23,24] and Wathra [33] studies. In Lissemore et al. [24], SICI was significantly reduced in older adults (both depressed and non-depressed) compared to younger adults; no significant ICF and CSP differences between groups were found. In Lissemore et al. [23], SICI, ICF, and CSP remained unchanged after 12 weeks of venlafaxine treatment. ICF remained unchanged post-treatment. Wathra described a substantial genetic contribution to SICI (particularly involving MARK4 and PPP1R37) and CSP (with EGFLAM) at the genome-wide level. Still, the study did not reveal strong genetic associations with ICF [33].

### 3.5. Efficacy and Neuroimaging

In our review, we examined the role of neuroimaging as a tool for assessing clinical efficacy. Among the 16 studies included in this analysis, only five reported the use of neuroimaging techniques. All five studies employed MRI or its variations. Four studies utilized MRI to enhance coil localization, while one study (Leblhuber [22]) used MRI to exclude circumscribed cerebral lesions. Two studies (Blumberger [21] and Goke [26]) investigated the same participant group, resulting in identical neuronavigation characteristics. Consequently, only the Blumberger [21] study will be considered in this section, as it was the first to describe this participant group. In his study, Trevizol compared two studies, only one of which included a description of neuronavigation, and the characteristics from this study will be detailed in this review [32].

In terms of neuronavigation, all three studies relied on MRI-based neuronavigation to determine stimulation targets for TMS, though some also referenced alternative methods. Blumberger [21] and Trevizol [32] described MRI-based neuronavigation for localizing the dorsolateral prefrontal cortex (DLPFC), while Trevizol [32] also mentioned a scalp-based 5 cm rule as an alternative approach. Quinn [28] incorporated both structural and functional MRI to achieve more precise neuronavigation. All studies targeted the left DLPFC for stimulation; however, their methods for localization differed: Blumberger defined the target based on anticorrelation with the subgenual cingulate cortex (SgCC), Trevizol [32] used either the 5 cm rule or MRI neuronavigation, focusing on Brodmann areas 9 and 46, and Quinn [28] applied resting-state fMRI connectivity analysis to define DLPFC targets based on functional anticorrelation with the SgCC. For coregistration, each study employed different systems: Blumberger [21] used the Visor 2 system (Advanced Neuro Therapeutics), Trevizol [32] utilized the miniBIRD system (Ascension Technology Group), and Quinn [28] employed the Localite neuronavigation system, using anatomical landmarks such as the nasion and bilateral tragus for alignment. In two studies, descriptions of stereotaxic coordinates or anatomical landmarks are provided. Blumberger [21] utilized the MNI-152 stereotaxic coordinate system, and Quinn employed the Brainnetome atlas and functional masks for neuronavigation [28].

As each study utilized different MRI scanners and imaging parameters, the description of the imaging protocols is heterogeneous. This variation in neuronavigation techniques, targeting strategies, and imaging methodologies highlights the diverse approaches used to enhance the precision and reliability of TMS in research settings.

## 4. Discussion

Available studies highlight that this treatment method stands out for its high safety profile, being free of significant adverse effects that could compromise patients’ quality of life. This aspect is particularly relevant from multiple perspectives, the first being treatment compliance. In the absence of side effects that might cause discomfort or impose significant restrictions, patients are far more likely to adhere consistently to the prescribed treatment, without interruptions caused by discouraging symptoms or absolute contraindications. Another critical perspective concerns the applicability of this treatment for elderly patients, a demographic group often faced with limited therapeutic options. Several factors contribute to these limitations, including pre-existing somatic conditions, potential drug interactions, and the natural physiological decline associated with aging. Under such circumstances, pharmacological treatment may pose significant challenges, either due to a restricted range of viable medications, an increased susceptibility to adverse reactions, or an inability to achieve optimal therapeutic dosages due to comorbidities. Given these constraints, transcranial magnetic stimulation (TMS) emerges as a promising alternative, characterized by its minimal side effects and contraindications. This innovative approach presents a compelling therapeutic option for elderly patients, offering substantial clinical benefits while mitigating the risks inherent in conventional pharmacological interventions [21,26,31].

Both patients who underwent pharmacological treatment and those who did not achieved, in most cases, favorable outcomes. This finding suggests that TMS is not merely an adjunctive therapy or a potentiator of pharmacological treatment in geriatric depression but can serve as a standalone therapeutic approach, yielding positive results. Moreover, a significant consideration is that TMS allows patients to reduce or discontinue pharmacological treatment altogether [22,25].

In this review, we evaluated the potential and adjustable parameters of protocols used in both controlled and uncontrolled trials that assessed the efficacy of TMS and its variations in late-life depression.

Transcranial magnetic stimulation (TMS) has been utilized since 1985 as a technique to assess cortical excitability [35]. TMS/rTMS protocols employ a time-varying magnetic field to generate an electrical current and depolarize the axons beneath a specialized coil placed against the head, non-invasively [35,36]. TMS is believed to exert long-lasting neuro-modulatory effects and hold significant potential for clinical applications [37].

Over the last 5 years, two meta-analyses, written by Cappon et al. [37] and Zhang et al. [38], have evaluated protocols that could be effective for the use of TMS in geriatric depression. Zhang’s [38] review found that active rTMS compared to sham stimulation produced significant antidepressant effects. Additionally, Zhang identified several factors associated with greater efficacy, including higher stimulation intensity, shorter treatment duration, a more significant total number of pulses, and the use of rTMS as a monotherapy. Cappon’s review [37] concluded that, despite most studies employing a pulse count lower than the FDA-prescribed protocol, TMS remains an effective treatment strategy for geriatric depression.

Our review differs from the aforementioned reviews in that we not only analyzed TMS but also examined studies incorporating variations in TMS, such as deep TMS and theta burst stimulation (TBS). This approach allowed us to specifically evaluate which type of TMS might be most beneficial for geriatric patients, providing a more targeted and clinically relevant analysis.

In the analysis of TMS/rTMS protocols, several emerging patterns were identified. First, there was consistency in the targeted brain areas, with the dorsolateral prefrontal cortex (DLPFC) being the predominant site for therapeutic interventions, whereas studies focusing on the motor cortex [20,23] primarily aimed to assess neurophysiological effects. Second, variations were observed in stimulation frequency and intensity. Higher frequencies (10–20 Hz) were predominantly used in excitatory protocols, while lower frequencies (3 Hz) were applied for inhibitory stimulation. The intensity of stimulation generally ranged between 110 and 120% of the resting motor threshold (RMT), except in studies that adjusted parameters for cortical excitability assessments. Lastly, study-specific adjustments were evident; some protocols increased the number of treatment sessions for non-remitters [21,32], while others [20,23] employed specialized stimulation paradigms such as paired associative stimulation (PAS), short-interval intracortical inhibition (SICI), intracortical facilitation (ICF), and cortical silent period (CSP).

From a clinical perspective, these findings offer a valuable framework for clinicians considering TMS/rTMS as a therapeutic strategy. The most effective protocols appear to involve high-frequency stimulation (10–20 Hz) applied to the left DLPFC, with session numbers adjusted based on patient response. Bilateral stimulation, as demonstrated in Blumberger et al., may be particularly beneficial for treating cases that are resistant to treatment by leveraging both excitatory and inhibitory mechanisms. Additionally, the potential benefits of more frequent stimulation sessions, such as twice-daily treatment [30], could be further investigated to determine whether they enhance therapeutic efficacy. Furthermore, while studies focusing on the motor cortex [23,31] provide essential insights into cortical plasticity, their direct clinical relevance remains limited.

Deep transcranial magnetic stimulation (TMS) is a novel FDA-approved therapeutic approach for treatment-resistant depression (TRD) [39] and is widely regarded as a safe and effective intervention [40]. While deep TMS has demonstrated significant success in treating TRD, there remains limited evidence regarding its efficacy in geriatric depression. In this review, we analyzed two studies that used deep TMS. While both studies share fundamental deep TMS principles, Kaster et al. [34]. pursued a more aggressive and extensive protocol, whereas Roth et al. [29]. adhered to a more standard approach. Kaster et al.’s [34] high-dose approach may offer greater neuroplastic benefits but at the cost of increased session length and potential side effects. Future research should investigate whether higher pulse numbers result in significantly better clinical outcomes compared to shorter protocols, such as those described by Roth et al. [29] Also, the coil selection is important, and the failure of the H1L coil in Kaster et al. [34] shows the importance of patient comfort in deep TMS. New coil designs should prioritize both effectiveness and tolerability to maximize adherence. The differences between these methodologies highlight ongoing debates in neuromodulation research regarding the optimal balance between stimulation intensity, treatment efficacy, and patient tolerability.

Regarding TBS, it is being increasingly utilized in both clinical and non-clinical research settings due to its ability to be delivered more rapidly and at lower intensities than conventional rTMS [41], while still inducing comparable or even enhanced neuroplastic effects relative to TMS [42].

The reduced duration of TBS protocols allows for multiple treatment sessions to be administered within a single day, facilitating more rapid therapeutic interventions [43]. This might enhance patient access to treatment while minimizing the time spent in clinical settings, thereby offering significant clinical benefits [44].

Blumberger [21] employs a more traditional bilateral stimulation paradigm characterized by a moderate pulse load and an extended treatment duration, incorporating adaptive titration to enhance tolerability. In contrast, Quinn [28] adopts an accelerated, high-dose protocol, which may be particularly beneficial for treatment-resistant cases but necessitates a substantial time commitment, with five sessions per day. The use of right-sided iTBS, as implemented in Quinn’s study, represents an emerging approach, especially in the treatment of depression with comorbid anxiety symptoms [28]. Future studies comparing these methods in a controlled setting could further validate their efficacy and optimal use cases.

Across the reviewed studies, remission and response rates following rTMS treatment for late-life depression varied considerably depending on the study design and stimulation parameters. Remission rates ranged from 20% to over 60%. In studies utilizing standard TMS or repetitive TMS (rTMS), Goke et al. [26] observed a 34.1% remission rate, identifying baseline depression severity and cognitive functioning as significant predictors of treatment response. Jodoin et al. [30] found a 63% remission rate among individuals aged 60 and above, while Leuchter et al. [25] reported remission in 36% of older adults (≥60 years), compared to 31% in younger adults (<60 years), highlighting potential age-related differences in treatment outcomes. Similarly, Trevizol et al. [32] reported a remission rate of 40%, while Blumberger et al. [21] noted a comparable outcome of 32.9% using a standard bilateral rTMS approach. Regarding theta burst stimulation (TBS), a more time-efficient protocol, this showed comparable outcomes: Blumberger et al. [21] reported a 35.4% remission rate with intermittent TBS (iTBS), while Quinn et al. [28] observed a lower remission rate of 20% using an accelerated iTBS protocol. In his study, Almheiri et al. [19] reported a 36% remission rate based on HDRS criteria, though the stimulation type was not explicitly detailed. These findings underscore the variability in treatment outcomes across different TMS modalities and highlight the importance of protocol selection in optimizing therapeutic efficacy for older adults with depression.

Regarding response rates, Roth et al. [29] again reported the most favorable outcomes, with a 69.2% response rate after 20 sessions and 79.4% after 30 sessions. Similarly, Quinn et al. [28] found a 52% response rate using an iTBS protocol, and Blumberger et al. [21] observed higher response rates for TBS (44.3%) compared to standard rTMS (32.9%). In Kaster et al. [34], active deep rTMS elicited significantly higher response rates (44% in the intention-to-treat sample and 55% in the per-protocol sample) compared to sham (18.5%). Leuchter et al. [25] reported that 58% of patients aged ≥60 were classified as responders on at least one outcome measure. Although response and remission rates were not consistently reported across all studies, the findings collectively suggest that both standard and accelerated TMS protocols can yield substantial clinical benefits.

The variability in remission and response rates across studies highlights the influence of stimulation parameters, treatment protocols, and patient characteristics on the efficacy of TMS in late-life depression. These findings suggest that both standard and patterned TMS approaches, including bilateral rTMS, TBS, and deep TMS, can offer clinically meaningful benefits, particularly when guided by individualized treatment planning and appropriate patient selection.

Although studies have demonstrated a significant deficit in neuroplasticity in individuals with depression [45], the question remains: from a clinical perspective, is it valuable to examine neuroplasticity in late-life depression?

Four studies investigating neuroplasticity were included, each employing a distinct methodological approach. Bhandari et al. [20] directly examined synaptic plasticity using paired associative stimulation (PAS), a protocol specifically designed to assess long-term potentiation (LTP)-like plasticity in the motor cortex. Lissemore et al. [24] explored cortical inhibition, facilitation, and plasticity in the context of venlafaxine treatment, utilizing PAS to determine whether antidepressant therapy influences neuroplasticity. In contrast, Lissemore et al. [24] focused on GABAergic inhibition, a key regulator of neuroplasticity, employing TMS measures—specifically, Short-Interval Intracortical Inhibition (SICI) and the cortical silent period (CSP)—to investigate the effects of aging and depression on inhibitory circuits. Wathra [33] investigated the genetic basis of TMS-induced cortical changes in LLD, highlighting the involvement of key neurotransmitter system genes and their connection to PAS [31].

While Bhandari et al. [20] and Lissemore et al. [23] explicitly assessed PAS-induced plasticity, Lissemore et al. [24] and Wathra [33] examined inhibition, which indirectly influences plasticity. Both Bhandari et al. [20] and Lissemore [23] found that PAS-induced plasticity did not significantly differ in late-life depression or change with antidepressant treatment, suggesting that age-related factors may exert a greater influence on plasticity than depression itself. Additionally, Lissemore [24] reported that aging reduces cortical inhibition, irrespective of depression status, further supporting the notion that neuroplasticity impairments in late-life depression may be primarily attributed to aging rather than the disorder itself.

In the context of neurotransmission and neuroplasticity in the treatment of geriatric depression using transcranial magnetic stimulation (TMS), Short-Interval Intracortical Inhibition (SICI) (GABA A-mediated inhibition), intracortical facilitation (ICF) (glutamate-mediated facilitation), and cortical silent period (CSP) (GABA B-mediated inhibition) were analyzed in two of the four studies on neuroplasticity, specifically Lissemore et al. [23] and Lissemore et al. [24]. The findings suggest that while aging reduces SICI (GABA A-mediated inhibition), depression itself does not exacerbate this decline. Furthermore, ICF, which reflects glutamatergic excitability, remains stable across aging and depression, indicating that neither condition significantly impacts this measure. Similarly, CSP, a marker of GABA B-mediated inhibition associated with longer inhibitory effects, is preserved in both aging and depression and remains unaffected by venlafaxine treatment. Overall, venlafaxine does not appear to significantly alter SICI, ICF, or CSP, further supporting the notion that age-related changes in cortical inhibition persist despite pharmacological intervention [23]. The lack of effect of venlafaxine on plasticity [23] suggests that pharmacological treatments might not directly impact neuroplasticity.

Regarding neuroplasticity and genetic analysis in Wathra’s study [33], the findings indicate a significant genetic association for SICI and CSP, particularly with MARK4, PPP1R37, and EGFLAM genes, suggesting a potential role for these genes in cortical inhibition and the regulation of the cortical silent period. In contrast, ICF did not reach genome-wide significance, implying that genetic influences on intracortical facilitation may be weaker or shaped by more complex mechanisms. From a clinical perspective, the overlap between SICI-associated genes and those previously linked to Alzheimer’s disease (AD) [46] suggests a possible connection between cortical inhibition and neurodegenerative processes, highlighting the need for further investigation.

All four studies agree that aging, rather than depression, plays a dominant role in neuroplasticity decline. Since PAS did not show significant differences in plasticity, TMS-based treatments for late-life depression may need alternative approaches. Given these findings, utilizing neuroplasticity assessments in the context of TMS for geriatric patients may have limited clinical utility, as aging appears to be the dominant factor influencing neuroplasticity decline rather than depression itself. Consequently, further research is warranted to explore alternative approaches that may enhance the effectiveness of TMS-based treatments for late-life depression and provide more actionable insights for clinical practice.

MRI has played an essential role in enhancing the precision of stimulation target localization when TMS is used as a therapeutic approach. Furthermore, it contributes to a deeper understanding of the neural mechanisms underlying depression and treatment response [47]. Moreover, the cost of an MRI scan is relatively low compared to the overall expense of a full course of TMS for depression, making it a cost-effective tool for optimizing treatment outcomes [48]. Accurate targeting may reduce the need for multiple treatment cycles, minimizing the risk of ineffective interventions. This is especially important for older adults, who often face mobility limitations; ensuring that TMS is effective from the first course of treatment can make the therapy more accessible by reducing the burden of repeated visits. In this context, the upfront investment in imaging could ultimately improve treatment efficiency, patient adherence, and outcomes in the geriatric population. The majority of the reviewed studies incorporating neuroimaging [21,28,32] utilized MRI-based neuronavigation to evaluate its potential clinical benefits. Regarding precision and practicality, Blumberger’s [21] reliance on anticorrelation with the subgenual cingulate cortex (SgCC) aligns with modern neuroscience models of TMS for depression, suggesting a strong neurobiological rationale for target selection [49]. Trevizol’s dual approach, incorporating both MRI-based localization and the 5 cm rule, highlights the trade-off between accuracy and accessibility. The 5 cm rule, while less precise, remains a commonly used method in clinical settings due to its simplicity and feasibility in environments without advanced neuronavigation. Quinn’s [28] use of resting-state fMRI connectivity analysis represents the most precise and individualized targeting method, as it accounts for functional brain variability between individuals. However, this resource-intensive approach requires advanced imaging and computational analysis, which may limit its widespread clinical use. Also, the use of different neuronavigation systems (Visor 2, miniBIRD, and Localite) [21,28,32] suggests a lack of consensus on the optimal approach for aligning stimulation targets. Therefore, greater efforts in standardization, methodological consistency, and clinical validation will be essential for optimizing the effectiveness and accessibility of TMS therapy.

### Clinical Implications and Patient-Centered Recommendations

Managing geriatric patients with depression presents a distinct set of challenges. This population often contends with complex clinical profiles that include multiple somatic comorbidities requiring pharmacological treatment, heightened sensitivity to therapeutic interventions, cognitive impairment, and reduced adherence to care plans. The benefits of incorporating patient-centered recommendations into decision-making are manifold. Additionally, it can help minimize the risk of overtreatment or undertreatment by ensuring that medical decisions are aligned with the patient’s values and life circumstances, leading to a more holistic and effective approach to care. These factors underscore the need for personalized treatment strategies when selecting neuromodulation therapies such as TMS. To address this, based on the data synthesized from the studies included in our review, we developed a clinical profile matrix aimed at guiding treatment decisions. This matrix (see Table 4) matches key patient characteristics—such as degree of treatment resistance, cognitive status, comorbidity burden, and urgency of response—with the TMS protocol most likely to offer benefit. For example, bilateral rTMS may be better suited for treatment-resistant depression, while theta burst stimulation (TBS) may be more appropriate for patients requiring rapid symptom relief or facing time constraints. Standard high-frequency rTMS remains a viable option for most cases, particularly when tolerability is a concern.

While this framework can assist clinicians in making individualized, evidence-informed treatment decisions for late-life depression, further clinical validation is necessary to strengthen its application in therapeutic planning.

## 5. Limitations

The limitations of our study were data heterogeneity, limitations related to study publication (studies with favorable effects are more often published), incomplete reporting of data, and incomplete procedure descriptions. Another limitation is the heterogeneity in study designs, as the included studies comprised both randomized controlled trials and retrospective analyses. Also, there is a language bias because only English studies were included.

Another limitation of this scoping review is the absence of a formal critical appraisal of the study quality. As a result, it is unable to assess the reliability or strength of the findings, which may affect the generalizability of the results. For instance, some studies may have small sample sizes, lack proper blinding, or have inadequate control groups, all of which could influence the conclusions regarding the effectiveness of TMS. Also, the absence of formal bias or risk assessment is considered a limitation of scoping methodology.

## 6. Conclusions

Transcranial magnetic stimulation (TMS) represents a viable and effective approach in the treatment of geriatric depression, demonstrating efficacy irrespective of concomitant pharmacological therapy. It is a safe intervention, with no significant adverse effects reported. Both high-frequency and low-frequency stimulation protocols can be employed, each contributing to measurable neuroplastic changes, particularly in cortical inhibition. Neurophysiological assessments underscore its impact on neural adaptability, further validating its therapeutic potential. Based on the available evidence, TMS is considered to be a reliable and well-tolerated modality for managing late-life depression across different protocols.

## Figures and Tables

**Table 1:** Study characteristics.

	Authors	Study	Country	Year	Key Words	Study Size	Outcome Measure
1	Almheiri et al. [19]	Effectiveness of Repetitive Transcranial Magnetic Stimulation in The Treatment of Depression in the Elderly: A Retrospective Natural Analysis	Germany	2023	late-life depression, geriatric depression, TMS, efficacy	78 pacients	HRDS21 MRI
2	Bhandari et al. [20]	Assessment of Neuroplasticity in Late-Life Depression with Transcranial Magnetic Stimulation	Canada USA	2023	late-life depression, geriatric depression, TMS, efficacy	56 pacients	MARDS
3	Blumberger et al. [21]	Effectiveness of Standard Sequential Bilateral Repetitive Transcranial Magnetic Stimulation vs. Bilateral Theta Burst Stimulation in Older Adults With Depression The FOUR-D Randomized Noninferiority Clinical Trial	Canada	2022	late-life depression, geriatric depression, TMS, efficacy	172 pacients	MADRAS, HRSD-17, QIDS-SR-16 MRI
4	F. Leblhuber et al. [22]	Treatment of patients with geriatric depression with repetitive transcranial magnetic stimulation	Austria	2019	late-life depression, geriatric depression, TMS	19 pacients	HAM-D MRI
5	Lissemore et al. [23]	Cortical inhibition, facilitation and plasticity in late-life depression: effects of venlafaxine pharmacotherapy	Canada	2020	late-life depression, geriatric depression, TMS	68 pacients	MADRS EMG
6	Lissemore et al. [24]	Reduced GABAergic cortical inhibition in aging and depression	Canada	2018	late-life depression, geriatric depression, TMS	92 pacients	HDRS-21 SSI
7	Leuchter et al. [25]	The effect of older age on outcomes of rTMS treatment for treatment-resistant depression	USA	2024	late-life depression, geriatric depression, rTMS	207 eldery pacients	HRDS-21 SCID MRI
8	Goke et al. [26]	Predictors of remission after repetitive transcranial magnetic stimulation for the treatment of late-life depression	Canada USA JAPAN	2024	Repetitive transcranial magnetic stimulation Late-life depression	164 pacients	HDRS-21, PHQ-9, BDI-II
9	Pan et al. [27]	The cognitive effects of adjunctive repetitive transcranial magnetic stimulation for late-onset depression: a randomized controlled trial with 4 week follow-up.	China	2023		58 pacients	IDS-SR, POMS, HDRS, PHQ
10	Quinn et al. [28]	Electric field distribution predicts efficacy of accelerated intermittent theta burst stimulation for late-life depression	USA	2023	late-life depression, geriatric depression, TMS, efficacy theta burst	25 pacients	IDS-C-30, Functional MRI, Electric field modeling
11	Roth et al. [29]	Never Too Late: Safety and Efficacy of Deep TMS for Late-Life Depression	USA	2024	late-life depression, geriatric depression, TMS, efficacy	247 pacients	MADRS
12	Jodoin et al. [30]	Safety and efficacy of accelerated rTMS protocol in elderly depressed unipolar and bipolar patients	USA	2018	late-life depression, geriatric depression, TMS, efficacy	19 eldery pacients	MADRAS
13	Wathra et al. [31]	Effect of prior pharmacotherapy on remission with sequential bilateral theta-burst versus standard bilateral repetitive transcranial magnetic stimulation in treatment-resistant late-life depression	UK	2023	late-life depression, geriatric depression, TMS, efficacy	164 pacients	MADRAS, HRSD-17, QIDS-SR-16 MRI
14	Trevizol et al. [32]	Unilateral and bilateral repetitive transcranial magnetic stimulation for treatment-resistant late-life depression	Canada	2018	late-life depression, geriatric depression, TMS, efficacy	43 pacients	MADRAS, HRSD-17, QIDS-SR-16 MRI
15	Wathra et al. [33]	Exploratory genome-wide analyses of cortical inhibition, facilitation, and plasticity in late-life depression	Canada	2023	late-life depression, geriatric depression, TMS, efficacy	79 pacients	MADRAS EMG
16	Tyler S. Kaster et al. [34]	Efficacy, tolerability, and cognitive effects of deep transcranial magnetic stimulation for late-life depression: a prospective randomized controlled trial	Canada	2018	late-life depression, geriatric depression, TMS, efficacy	80 pacients	MADRAS EMG

**Table 2:** TMS protocol characteristics.

Study	Device	Target Area	Frequency	Intensity of RMT	Protocol	Remission Rate	Response Rate
Leblhuber et al. [22]	Theracell apparatus (Guth Meditec, Salach, Germany)	Bilateral PFC	3 Hz	0.08 T	30 min per session 10 sessions, 2 weeks HAMD-7	Not reported	Not reported
Jodoin et al. [30]	MagPro X100 (MagVenture, Farum, Denmark)	DLPFC	20 Hz	110%	15 min per session (3000 pulses/session), 20 to 30 sessions, twice daily, 3–5 days/week MADRS, HAM-A	63%	73.7%
Blumberger et al. [21]	MagPro X100 (MagVenture)	Bilateral DLPFC	1 Hz and 10 Hz	120%	600 pulses over 10 minutes to the right DLPFC, followed by 3000 pulses: 4 s on, 26 s off over 37.5 min to the left DLPFC; 20 initial daily sessions over 4 weeks (an additional 10 daily sessions over 2 additional weeks if they did not achieve remission) MADRS, HRSD-17, QIDS-SR-16	21.4–32.9%	29.6–35.7
Bhandari et al. [20]	Magstim 200 (Magstim Company Ltd., Sheffield, UK) (Two stimulators)	Left motor cortex	Not specified, using PAS protocol	Adjusted for test stimulus	Focus on motor cortex with PAS protocol MADRS, MMSE, CIRS-G	Not reported	Not reported
Trevizol et al. [32]	Magventure RX-100 (MagVenture)	DLPFC	10 Hz for high frequency unilateral, 1 Hz for bilateral	120%	15 sessions initially, with an additional 15 sessions if non-remitting 5 sessions/week; 3 weeks HDRS	40% bilateral/0% unilateral/ sham 0%	45 bilateral/0% unilateral/sham 16.7%
Leuchter et al. [25]	MagPro X100, Magstim Horizon, Magstim Super Rapid2, or Neurostar	Left DLPFC	10 Hz	120%	30 sessions beginning with HFL IDS, POMS, PHQ-9, HDRS	25–27%	25–36%
Pan et al. [27]	Magstim (eight-coil device)	DLPFC	10 Hz	120%	20 min per session (800 pulses) 4 weeks HDRS, RBANS	Not reported	Not reported
Lissemore et al. [23]	Magstim 200 (Two stimulators, Bistim module)	Left motor cortex	Not specified, uses various TMS paradigms (SICI, ICF, CSP, PAS)	80%/140%	Assesses cortical physiology pre- and post-venlafaxine treatment with various TMS paradigms. MADRS	Not reported	Not reported
Lissemore et al. [24]	Magstim 200 (Two stimulators, Bistim module)	Left motor cortex	Not specified (focus on SICI, ICF, CSP)	80%/140%	Measures SICI, ICF, CSP, and PAS with precise coil positioning for cortical excitability. MADRS, CIRS-G	Not reported	Not reported
Wathra et al. [31]	Magstim 200 (Two stimulators, Bistim module)	Left motor cortex	Not specified (focus on PAS)	80%/140%	Uses PAS with sensory threshold for peripheral nerve stimulation and MEP amplitude measures. MADRS	Not reported	Not reported

PFC = prefrontal cortex, T = tesla, DLPFC = dorsolateral prefrontal cortex, Hz = Herz, PAS = paired associative stimulation, RMT = resting motor threshold, HFL = high frequency left, SICI = short-interval intracortical inhibition (SICI), ICF = intracortical facilitation, CSP = cortical silent period, MEP = Motor evoked potential, HAMD-7 = The 7-Item Hamilton Depression Rating Scale, MADRS = Montgomery–Åsberg Depression Rating Scale, HAM-A = Hamilton Anxiety Rating Scale, HDRS = Hamilton Depression Rating Scale, QIDS-SR-16 = Quick Inventory of Depressive Symptomatology Self Report, MMSE = Mini Mental State Examination, CIRS-G scale = Cumulative Illness Rating Scale-Geriatric, IDS = Inventory of Depressive Symptomatology, POMS = Profile of Mood States, PHQ-9 = Patient Depression Questionnaire, RBANS = Repeatable Battery for Neuropsychological Status.

**Table 3:** Neuroplasticity assessment characteristics.

Study	Therapeutic Approach	Additional Group Comparison	Neuroplasticity Measures	Cortical Target	Nerve Targeted	Stimulation Parameters	Remission Rate	Response Rate
Bhandari et al. (2018) [20]	Directly measures PAS-induced synaptic plasticity (LTP-like effects)	Age-matched healthy controls	PAS-LTP (MEP potentiation after PAS)	Left motor cortex	Right median nerve	PAS: 180 pairs, 25 ms delay, 300% sensory threshold	Not reported	Not reported
Lissemore et al. (2020) [23]	Examines whether venlafaxine treatment affects cortical plasticity	No	PAS-LTP, SICI, ICF, CSP	Left motor cortex	Right median nerve	PAS: 180 pairs, 25 ms delay, 3× participant’s sensory threshold	Not reported	Not reported
Lissemore et al. (2018) [24]	Examines GABAergic inhibition (which regulates neuroplasticity)	Age-matched and younger healthy controls, younger depressed adults	SICI (GABA A), CSP (GABA B), ICF (glutamate)	Left motor cortex	None (Cortical inhibition focus)	TMS inhibition protocols: SICI (2 ms), CSP (140% RMT), ICF (10 ms)	Not reported	Not reported
Wathra et al. (2023) [33]	Examines the genetic basis of TMS-induced cortical changes in LLD, focusing on BDNF polymorphisms and key neurotransmitter system genes.	No	SICI, CSP, ICF	Left motor cortex	Right median nerve	PAS: 180 pairs, 25 ms delay, no participant’s sensory threshold provided (focus on motor inhibition)	Not reported	Not reported

PAS = paired associative stimulation, LTP = long-term potentiation, MEP = motor-evoked potentials, SICI = Short-interval intracortical inhibition, CSP = cortical silent period, ICF = intracortical facilitation, GABA A, B = Gamma-aminobutyric-acid A, B, LLD = late life depression, BDNF = brain-derived neurotrophic factor.

**Table 4:** Patient-centered recommendations.

Patient Profile	Recommended Protocol	Rationale
Mild-to-Moderate Depression, No Major Comorbidities	Standard high-frequency (10–20 Hz) rTMS over left DLPFC	Most common, well-tolerated protocol; strong evidence base; good balance of efficacy and safety
Severe Depression, Treatment-Resistant	Bilateral rTMS (e.g., 1 Hz right + 10 Hz left) or Deep TMS	Higher remission rates observed; bilateral protocols may better modulate network-level dysfunction. Deep TMS (e.g., BrainsWay H1 coil) showed up to 60% remission with longer duration
Polypharmacy or Medication Intolerant	Monotherapy TMS (without concurrent pharmacotherapy)	Several studies show clinical improvement without meds, making TMS suitable as a standalone treatment
Time-Constrained or Inpatient Setting	Accelerated TBS (iTBS, cTBS protocols)	Rapid delivery (3–5 min per session), allowing multiple sessions/day; suitable for faster symptom reduction
Comorbid Cognitive Decline or Mild Cognitive Impairment	Protocols with neurocognitive monitoring (e.g., Pan [27], Jodoin [30])	Some protocols included cognitive batteries (RBANS, MMSE); iTBS and high-frequency rTMS generally well-tolerated cognitively
Patients with High Sensitivity to Pain or Discomfort	Standard rTMS with moderate intensity (≤110% RMT)	Avoid deep TMS or high-pulse protocols like H1L coil (Kaster [34]); standard coils better tolerated
Patients with Poor Treatment Adherence	Protocols with short duration or low side effects (e.g., iTBS)	Shorter protocols improve adherence. Adverse effects are minor and transient across most protocols
Interest in Cognitive Enhancement or Neuroplasticity	PAS-enhanced TMS or protocols with motor cortex targets	Some studies (e.g., Bhandari [20], Lissemore [23,24] explore cortical plasticity changes using paired associative stimulation. Though clinical relevance remains limited.

## Data Availability

Data are contained within this article.
